# Neoadjuvant bevacizumab and chemoradiotherapy in locally advanced rectal cancer: early outcome and technical impact on toxicity

**DOI:** 10.1186/1477-7819-12-329

**Published:** 2014-11-06

**Authors:** Chia-Chun Wang, Jin-Tung Liang, Chiao-Ling Tsai, Yu-Hsuan Chen, Yu-Lin Lin, Chia-Tung Shun, Jason Chia-Hsien Cheng

**Affiliations:** Department of Oncology, National Taiwan University Hospital Yun-Lin Branch, Yun-Lin, Taiwan; Graduate Institute of Epidemiology and Preventive Medicine, College of Public Health, National Taiwan University, Taipei, Taiwan; Department of Surgery, National Taiwan University Hospital, Taipei, Taiwan; Department of Oncology, National Taiwan University Hospital, Taipei, Taiwan; Department of Pathology, National Taiwan University Hospital, Taipei, Taiwan; Graduate Institute of Oncology, National Taiwan University College of Medicine, Taipei, Taiwan; Division of Radiation Oncology, Department of Oncology, National Taiwan University Hospital, No.7, Chung Shan S. Rd., Taipei, 10002 Taiwan

**Keywords:** bevacizumab, prone position, radiotherapy, rectal cancer, volumetric modulated arc therapy

## Abstract

**Background:**

We aimed to evaluate early clinical and pathological results for treating locally advanced rectal cancer with bevacizumab and neoadjuvant concurrent chemoradiotherapy using the technique of prone-position volumetric modulated arc therapy and to compare the toxicity of volumetric modulated arc therapy with that of supine-position four-field box radiotherapy.

**Methods:**

Twelve patients with stage IIA to IVA rectal adenocarcinoma, treated with neoadjuvant concurrent chemoradiotherapy (45 Gy in 25 fractions to the rectal tumor and pelvic lymphatics) and bevacizumab, were prospectively enrolled. Chemotherapy included FOLFOX (leucovorin, fluorouracil, and oxaliplatin) (*n* =11) and 5-fluorouracil (*n* =1). All patients received prone-position volumetric modulated arc therapy. A historical cohort treated with supine-position box radiotherapy, including six other patients treated with bevacizumab-based concurrent chemoradiotherapy in our hospital, was used for comparison. Setup errors, toxicities, and potential biomarkers were evaluated.

**Results:**

All patients completed neoadjuvant concurrent chemoradiotherapy and underwent total mesorectal excision. Four (33.3%) patients had pathological complete response. Significantly more grade 2 or 3 diarrhea was associated with the supine-box technique (5/6 versus 2/12, *P* =0.01). The magnitude of setup errors was similar between the supine-box and prone volumetric modulated arc therapy techniques. The estimated 2-year survival and 2-year failure-free survival rates were 100% and 72.9% in the prone volumetric modulated arc therapy group and 66.7% and 66.7% in the supine box group, respectively.

**Conclusions:**

The early clinical outcome has been encouraging. Volumetric modulated arc therapy in prone-positioned patients was technically advantageous and reduced bowel toxicity.

## Background

Neoadjuvant concurrent chemoradiotherapy has become the standard for locally advanced rectal cancer care. Previous studies showed that preoperative concurrent chemoradiotherapy reduced the recurrence rate and increased the sphincter preservation rate, when compared with postoperative concurrent chemoradiotherapy [1, 2]. The use of various chemotherapeutic agents and targeted therapies has been investigated in conjunction with neoadjuvant concurrent chemoradiotherapy. Nevertheless, treatment-related toxicities have limited the success of these combination therapies.

Bevacizumab, an anti-vascular endothelial growth factor monoclonal antibody, is widely used in metastatic colorectal cancer [3, 4]. Although it augments the therapeutic effect of ionizing radiation [5, 6], bevacizumab has been shown to have significant gastrointestinal toxicities when combined with radiotherapy [7]. In several studies evaluating the efficacy and safety of concurrent chemoradiotherapy combined with bevacizumab for rectal cancer, a favorable outcome with acceptable toxicities was reported for capecitabine or fluorouracil with bevacizumab in neoadjuvant concurrent chemoradiotherapy [8, 9], while some excessive toxicities were still reported for capecitabine and bevacizumab or bevacizumab, oxaliplatin and 5-fluorouracil in neoadjuvant concurrent chemoradiotherapy [10, 11].

Intensity-modulated radiation therapy has gradually replaced traditional four-field box radiotherapy in rectal cancer treatment because it improves dose distribution and reduces bowel exposure [12, 13]. Volumetric modulated arc therapy, a recently developed technique involving arc intensity-modulated radiation therapy delivery, has been shown to further improve dose conformity and organ sparing [14, 15]. Prone positioning on a belly board has effectively reduced bowel doses in radiotherapy for pelvic malignancies [13, 16–18]. The clinical outcome of prone-position volumetric modulated arc therapy has not been assessed for rectal cancer. The aims of this study were, firstly, to evaluate early pathological and clinical outcome in patients with locally advanced rectal cancer treated with combined neoadjuvant bevacizumab and concurrent chemoradiotherapy using prone-position volumetric modulated arc therapy and, secondly, to compare the treatment-related toxicity of the supine-position box and prone-position volumetric modulated arc therapy techniques.

## Methods

### Patients

Eligible patients had histologically confirmed rectal adenocarcinoma within 15 cm above the anal verge. Pretreatment staging workup included complete physical examination; chest X-ray; colonoscopy; computed tomography or magnetic resonance imaging of the abdomen and pelvis; and optional positron emission tomography. Endorectal ultrasound was obtained for tumor (T) and node (N) stages in patients by computed tomography of the pelvis. Patients with T3 or T4 or N1 or N2 disease were included. An Eastern Cooperative Oncology Group performance status of 0 or 1 was required. All patients gave their informed consent.

### Radiotherapy technique

Treatment was delivered with a 10 MV photon beam. We used an Elekta Synergy® linear accelerator upgraded with a volumetric modulated arc therapy function (Elekta Oncology System Ltd., Crawley, West Sussex, UK) for all patients receiving volumetric modulated arc therapy. For the six patients receiving three-dimensional therapy, we used the Elekta Synergy® linear accelerator for five patients and a Siemens PRIMUS linear accelerator (Siemens Medical System Inc., Concord, CA, USA) for one patient. The total dose was 45 Gy, given in 25 fractions of 1.8 Gy (one fraction per day; five fractions per week). Before March 2010, the four-field box technique was used in the supine position. After March 2010, the volumetric modulated arc therapy technique was used in the prone position on a belly board. Our institutional review board approved the protocol of this study. The volumetric modulated arc therapy plans were optimized using Pinnacle^3^ version 9.0 planning system software (ADAC Laboratories, Philips Medical Systems, Milpitas, CA, USA). We used a collapse cone convolution algorithm and a grid size of 4 mm [19]. Cone-beam computed tomography or orthogonal views were required for image guidance in all patients.

### Simulation and target definition

Patients were instructed to maintain a tolerably full bladder. A belly board was used to move the small bowels away from the radiation field for patients receiving volumetric modulated arc therapy. A planning computed tomogram (maximum slice thickness, 5 mm) was acquired using the same immobilization technique as for treatment.

The gross tumor volume was defined as all known gross disease, as determined from a combination of imaging studies. The clinical target volume was defined as the gross tumor volume plus areas considered at significant risk of microscopic disease. The clinical target volume for a T3 tumor included all gross disease (rectal and nodal) as well as the internal iliac lymph nodes and the mesorectum (perirectal fat and presacral space). The clinical target volume for a T4 tumor included the same structures as defined in the clinical target volume for a T3 tumor and also the external iliac lymph nodes. To be more specific, the rectal clinical target volume was defined as the rectal gross tumor volume plus a 1.5 cm margin radially and a 2.5 cm margin craniocaudally. The nodal clinical target volume was defined as the nodal gross tumor volume plus a 1.5-cm symmetrical expansion of this volume. The clinical target volumes around vessels were defined as vessel volumes plus a 0.7-cm expansion of these volumes. The presacral lymphatic clinical target volume was contoured from mid S1 to S5. The planning target volume was the clinical target volume with a 5-mm margin.

The organs at risk, including the small bowel, bladder, prostate, uterus, and bilateral femurs were contoured accordingly. When the clinical target volume overlapped the small bowel, the clinical target volume was manually trimmed to reduce the exposure of the small bowel. The planning target volume was not modified when it overlapped the organs at risk.

### Treatment parameters

For four-field box radiotherapy, the prescribed dose was delivered to the field isocenter. The dose weightings (ratios) for the anterior-posterior opposed beams and bilaterally opposed beams were either 1:1 or 6:4. For volumetric modulated arc therapy, ten patients had two partial arcs and two patients had three partial arcs. We used the volumetric modulated arc therapy technique with partial arcs by excluding gantry angles between 120° and 240° to treat patients in the prone position on a belly board, mainly to reduce the radiation dose to the urinary bladder and small bowels. This planning method helps achieve conformity of targets and simultaneously spares the critical organs.

### Chemotherapy

All patients received bevacizumab (5 mg/kg given every 2 to 3 weeks, for a median of five cycles) and oxaliplatin, 5-fluorouracil, and leucovorin (FOLFOX: 5-fluorouracil 1600 to 2800 mg/m^2^, oxaliplatin 40 to 85 mg/m^2^, and leucovorin 300 mg/m^2^; every 2 to 3 weeks for a median of five cycles), except that one patient was only given 5-fluorouracil, owing to old age.

### Surgery

Total mesorectal excision was performed 6 to 8 weeks after the completion of concurrent chemoradiotherapy. Rectum and pelvic lymphatics were removed. The surgical technique was either abdominoperineal resection or low anterior resection. Total mesorectal excisions were conducted laparoscopically in 13 patients.

### Measurement of setup errors

Image-guided tools were used to measure the absolute values of displacements in the superior-inferior, left-right, and anterior-posterior directions. For patients receiving volumetric modulated arc therapy, we used cone-beam computed tomography with an Elekta Synergy® X-ray volume imaging system (Elekta Oncology System Ltd., Crawley, West Sussex, UK) in all patients to evaluate setup errors. Approximately 650 projections were collected during a 360° rotation of the gantry in a clockwise direction. All cone-beam computed tomograms were compared with simulation computed tomograms using the pelvic bony structure as a reference. Electronic portal images were used in those receiving three-dimensional conformal radiotherapy. We compared the electronic portal images with digitally reconstructed radiographs from computed tomograms, by using the pelvic bony landmarks including the sacral edge and the pelvic ring, to measure and correct setup errors. With the average displacements from each patient, a group average was calculated for each of these three directions.

### Toxicity and outcome assessments

Toxicity was evaluated weekly during concurrent chemoradiotherapy and was graded using *Common Terminology Criteria for Adverse Events*, version 4.0. The primary endpoints were toxicity and pathological response, including pathological complete response and downstaging rates. Secondary endpoints included failure-free survival and overall survival. Survival was calculated from the first day of radiotherapy to the dates of the last follow-up visit or any recurrence (for failure-free survival) and death (for overall survival).

### Immunohistochemical staining

The pre-concurrent chemoradiotherapy biopsy sample and post-concurrent chemoradiotherapy surgical tissues were stained immunohistochemically using monoclonal antibodies against CD34 (Dako Denmark A/S, Glostrup, Denmark), Akt (Cell Signaling Technology Inc., Danvers, MA, USA), epidermal growth factor receptor (Dako Denmark A/S, Glostrup, Denmark), and vascular endothelial growth factor receptor 2 (Cell Signaling Technology Inc., Danvers, MA, USA). The slices were reviewed by an experienced pathologist at our institution.

### Statistical analysis

The statistical analysis was conducted using IBM SPSS statistics v.20.0 (IBM Corp., Armonk, NY, USA). Fisher’s exact test was used for the analysis of contingency tables. Student’s *t* test was used to evaluate differences in the volume receiving a certain dose or magnitude of setup errors between the supine- and prone-position groups. Kaplan-Meier analysis was used for survival comparisons. Differences were considered significant at *P* <0.05.

## Results

### Patient characteristics

Twelve patients with stage IIA to IVA rectal adenocarcinoma treated with neoadjuvant concurrent chemoradiotherapy and bevacizumab from March 2010 to March 2012 were prospectively enrolled in this study and were treated in the prone position with volumetric modulated arc therapy (prone volumetric modulated arc therapy). We also retrospectively collected details of all patients treated with neoadjuvant concurrent chemoradiotherapy and bevacizumab before the new technique was used and identified six patients, who all received four-field box radiotherapy in the supine position (supine box). The median follow-up time was 22.4 and 34.2 months in the prone volumetric modulated arc therapy and supine box cohorts, respectively. Patient characteristics (Table 1) included clinical T3 or T4 disease (*n* =17), clinical nodal involvement (*n* =14), and tumor location within 5 cm above the anal verge (*n* =6). The average delivery time of volumetric modulated arc therapy was 285 ± 46 s. The group averages of displacements in the superior-inferior, left-right, anterior-posterior directions (0.27 ± 0.09 cm, 0.20 ± 0.10 cm, and 0.34 ± 0.15 cm in the prone-position volumetric modulated arc therapy group, and 0.16 ± 0.18 cm, 0.14 ± 0.09 cm, and 0.24 ± 0.17 cm in the supine-position box group, respectively) were not significantly different between the two groups (*P* =0.12, 0.25, and 0.22, respectively).

**Table 1:** **Patient characteristics in different cohorts**

Characteristics	Prone volumetric modulated arc therapy group (n = 12)	Supine box group (n = 6)
Age (years)		
Median	52.5	57.5
Range	38 to 72	53 to 71
Sex		
Male	7	5
Female	5	1
Clinical tumor stage		
T2	1	0
T3	8	5
T4	3	1
Clinical node stage		
N0	2	2
N1	2	4
N2	8	0
Clinical stage		
IIA	2	2
IIIB	6	2
IIIC	4	0
IVA	0	2
Distance from the anal verge		
< 5 cm	5	1
5 to 10 cm	7	4
≥ 10 cm	0	1
Chemotherapy		
Bevacizumab + FOLFOX (leucovorin, fluorouracil, and oxaliplatin)	11	6
Bevacizumab +5-fluorouracil	1	0
Surgical type		
Lower anterior resection	9	3
Abdominoperineal resection	3	1

### Toxicities

**Figure 1:**
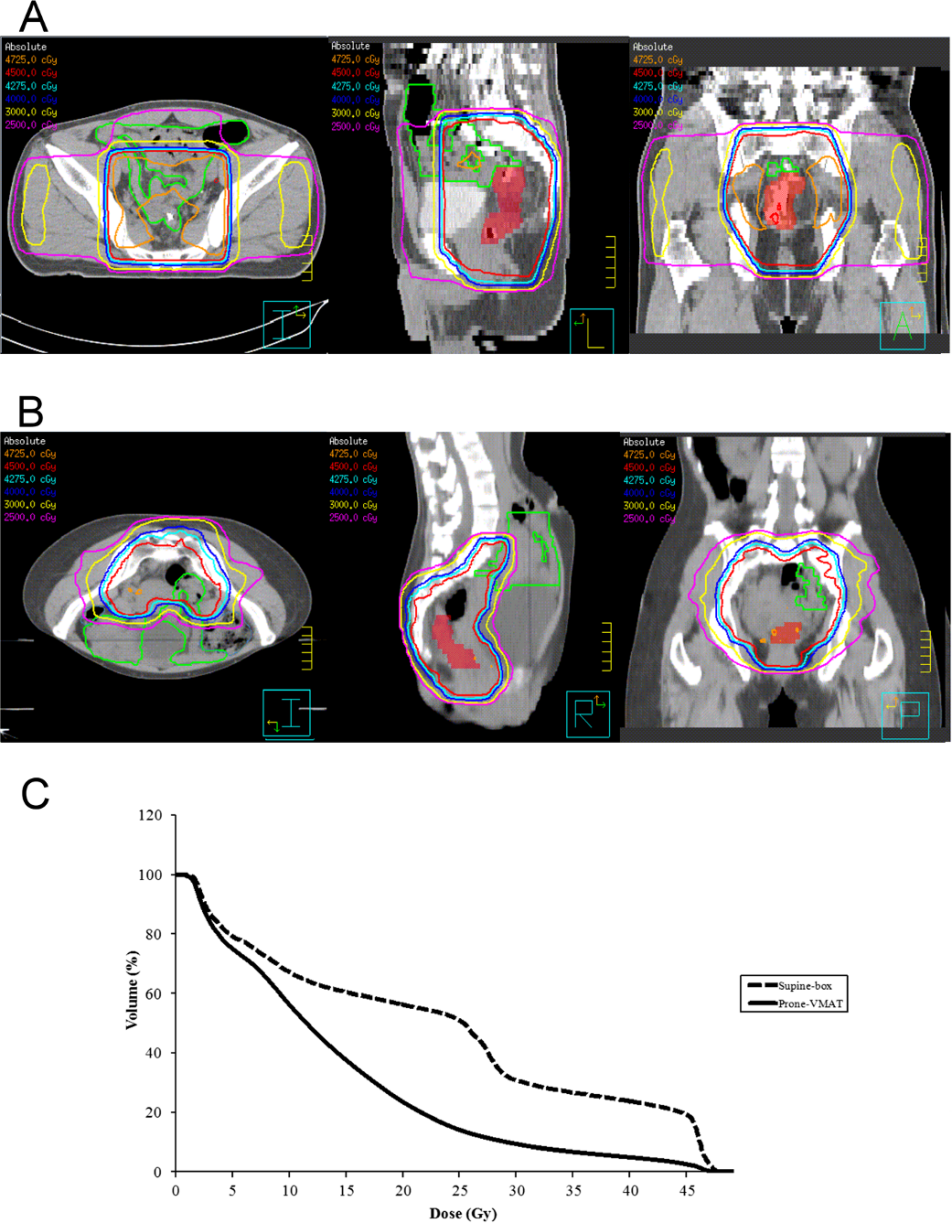
**Dose distributions.** Axial (left), sagittal (middle), and coronal (right) views of two representative patients treated by **(A)** supine-position four-field box radiotherapy and **(B)** prone-position volumetric modulated arc therapy. Small bowels are contoured in green and gross tumor volume is colored red. **(C)** Average dose-volume histogram of small bowels from patients by supine-box technique (dashed line) and prone volumetric modulated arc therapy technique (solid line).

The most common acute toxicities during concurrent chemoradiotherapy (Table 2) were grade 1 or 2 anal pain and anemia. Grade 3 toxicity was observed in three patients (two patients with neutropenia and one with diarrhea). There was no febrile neutropenia that required hospitalization. No difference in treatment-related toxicity (except bowel toxicity) was evident between the supine-position box and prone-position volumetric modulated arc therapy groups. Five of the six (83%) patients in the historical cohort and two of the twelve (17%) patients in the prone volumetric modulated arc therapy group experienced grade ≥2 diarrhea (*P* =0.01). Bowel toxicity was probably reduced by the significantly smaller bowel volume irradiated. The between-group differences in average small bowel volumes receiving 35 Gy, 40 Gy, and 45 Gy were all significant (*P* =0.005, 0.002, and 0.0006, respectively). The dose distribution (one representative patient from each group) and the average dose-small bowel volume histogram for each group are shown in Figure 1.

**Table 2:** **Acute toxicities during concurrent chemoradiotherapy**

	Prone volumetric modulated arc therapy group			Supine box group		
	Grade 1	Grade 2	Grade 3	Grade 1	Grade 2	Grade 3
Anal pain	6	3	0	0	3	0
Diarrhea	0	2	0	0	4	1
Anemia	3	3	0	5	1	0
Neutropenia	3	0	2	2	0	0
Thrombocytopenia	2	0	0	3	0	0

### Surgical outcome

All 12 patients receiving prone volumetric modulated arc therapy treatment completed neoadjuvant concurrent chemoradiotherapy with bevacizumab and received total mesorectal excision. Two patients in the supine box cohort refused surgery. Pathological responses, including a pathological complete response rate of 33.3%, are shown in Table 3. Postoperative complications included one perianal abscess formation and one postoperative wound infection, and both resolved after treatment. There was no between-cohort difference in pathological response (1/4 in the supine box group versus 4/12 in the prone volumetric modulated arc therapy group, *P* =1.00). The hospital stay was 7 to 24 days (median, 11).

**Table 3:** **Pathological stage and response**

Outcome	Prone volumetric modulated arc therapy group (n = 12)	Supine box group (n = 4)
Pathological stage		
T0N0	4	1
T2N0	3	1
T3N0	2	2
T2N2	2	0
T3N2	1	0
T-downstaging		
Yes	9	3
No	3	1
N-downstaging		
Yes	7	3
No	3	0

### Survival

**Figure 2:**
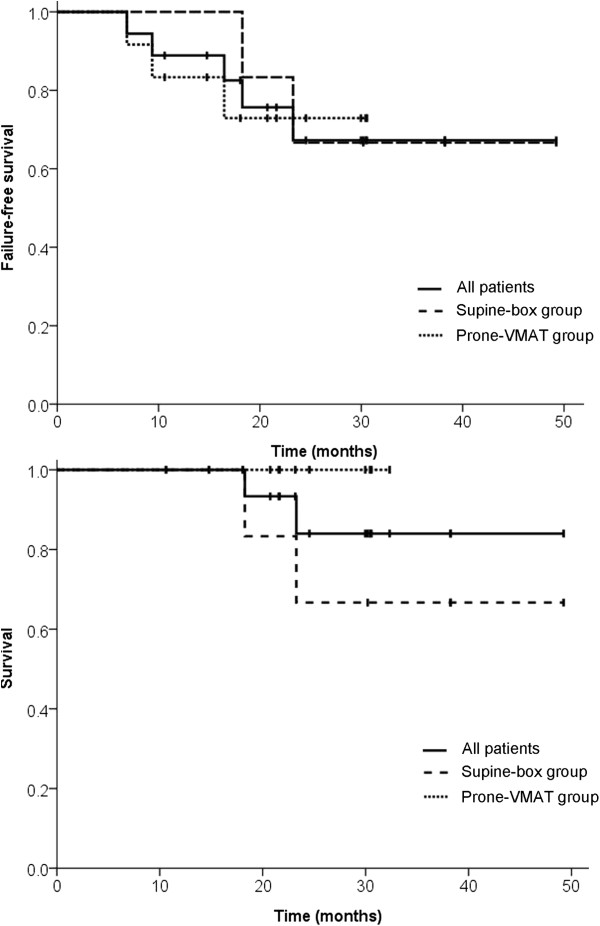
**Kaplan**-**Meier estimates of failure**-**free survival and overall survival of patients with locally advanced rectal cancer undergoing neoadjuvant bevacizumab and chemoradiotherapy.** The solid line, dashed line, and dotted line represent all 18 patients, 6 patients in the supine box group, and 12 patients in the prone volumetric modulated arc therapy group, respectively.

Kaplan-Meier estimates of failure-free survival and overall survival rates are shown in Figure 2. The median estimated survival rate was not reached in both cohorts. The estimated 2-year survival and 2-year failure-free survival rates were 100% and 72.9% in the prone volumetric modulated arc therapy group and 66.7% and 66.7% in the supine box group, respectively. In the supine box cohort, two patients with stage IVA disease were dead at 18.3 and 23.3 months. In the prone volumetric modulated arc therapy group, two patients had locoregional recurrence and three patients, including those two with locoregional recurrence, had distant metastasis. All the failures occurred within 2 years.

### Immunohistochemical staining

Owing to the few pathological samples for immunohistochemical study, we combined the two cohorts for analysis. Postoperative pathology specimens were available in thirteen patients, while nine of them also had pre-concurrent chemoradiotherapy specimens. Expression of CD34 was upregulated in the tumor area after concurrent chemoradiotherapy in six of seven patients with less than pathological complete response and zero of two patients with pathological complete response (*P* = 0.08). Akt was overexpressed in six of nine patients with both pre-concurrent chemoradiotherapy and postoperative specimens. The expression of vascular endothelial growth factor receptor 2 and epidermal growth factor receptor did not differ significantly between pre-concurrent chemoradiotherapy and postoperative specimens.

## Discussion

In this prospective study combined with historical comparison, we proposed the technical advantage of using volumetric modulated arc therapy in patients prone-positioned on a belly board, and reported the early pathological and clinical results for combined treatment with bevacizumab and neoadjuvant concurrent chemoradiotherapy in locally advanced rectal cancer. We demonstrated that the prone volumetric modulated arc therapy technique resulted in less toxicity and possibly better pathological response and clinical outcome. To our knowledge, this is the first report on survival outcome and local or distant disease control by neoadjuvant volumetric modulated arc therapy in rectal cancer. This technique allows the concomitant use of oxaliplatin and bevacizumab, which were previously shown to improve pathological response in randomized trials. Although acceptable, the magnitude of setup errors required the use of image-guided procedures for treatment.

A previous study using preoperative bevacizumab with FOLFOX and supine box radiotherapy found postoperative complications including delayed healing, leak or abscess, ischemic colonic reservoir, and fistula in 36% of patients [10]. Our patients had a much lower complication rate (only 2 of 12 patients) and a more reasonable postoperative hospital stay (7 to 24 days). An Austrian group terminated the patient accrual of a phase II trial combining bevacizumab and capecitabine with three-field radiotherapy because two of eight patients (25%) experienced grade 3 diarrhea and intestinal bleeding [11]. In our study, bowel toxicity was much reduced; the mean small bowel volume receiving more than 45 Gy was as small as 24.8 ml. Moreover, the toxicity profile of our prone volumetric modulated arc therapy approach was much lower than that in previous studies and that of our supine box approach. This improvement is beneficial for patients with locally advanced rectal cancer, as well as selected stage IV patients [20]. Of note, neoadjuvant chemotherapy without routine use of radiotherapy was found in a pilot study, and was well tolerable with no radiation-related side effects [21].

Compared with box techniques, step-and-shoot intensity-modulated radiation therapy provides superior planning target volume coverage, dose homogeneity, and conformity; it decreases the volume of small bowel exposed to radiation, but requires a longer delivery time [12]. Staying in the prone position on a belly board for such a long treatment period might increase the magnitude of setup errors. Volumetric modulated arc therapy is capable of dose delivery in a shorter timeframe. In our study, the average delivery time was as short as 285 s, suggesting a lower intrafraction motion error.

It was shown that a collapsed cone convolution algorithm might underestimate the dose in water medium after the photon beam traversed an air gap [19]. Special attention was suggested for possible setup errors and internal organ motion. We did not override the density of rectal gas (if present) during the planning phase. According to our imaging guidance protocol in this study, we used cone-beam computed tomography frequently to monitor for setup error and internal organ motion. We did not observe much change in rectal lumen during the radiotherapy course.

Volumetric modulated arc therapy provided superior target coverage compared with three-dimensional conformal radiotherapy and even step-and-shoot intensity-modulated radiation therapy in two studies on rectal cancer and anal cancer [14, 15]. Both studies used the supine position. Our volumetric modulated arc therapy approach used the prone position, which further reduced the volume of small bowel exposed to radiation. One dosimetric study from Italy showed that the same volumetric modulated arc therapy approach (compared with three- or four-field radiotherapy) reduces small bowel exposure to radiation, and (compared with intensity-modulated radiation therapy) shortens treatment time [22]. However, unlike our study, this study did not investigate clinical outcome (toxicities, magnitude of setup errors, pathological responses, or survivals).

Compared with the traditional four-field box technique, intensity-modulated radiation therapy or volumetric modulated arc therapy irradiates a smaller pelvic area, mainly the lymphatic region and the peritumoral area. The issue of radiotherapy precision by intensity-modulated radiation therapy or volumetric modulated arc therapy has not been well addressed in rectal cancer. Pelvic lymphatics are technically challenging sites to irradiate using intensity-modulated radiation therapy or volumetric modulated arc therapy. Our use of image guidance and the corresponding data on setup errors support our claim of accurate volumetric modulated arc therapy delivery. Our results showed only three patients with pathologically involved lymph nodes after concurrent chemoradiotherapy, and seven of ten patients with initial clinical node-positive disease had an N-downstaging response after volumetric modulated arc therapy.

Our study revealed increased expression of CD34 (possibly associated with increased microvessel density) after concurrent chemoradiotherapy, and increased expression of Akt before and after concurrent chemoradiotherapy. The increased expression of Akt after radiotherapy is compatible with previous reports [23–25]. The data on CD34 expression after treatment with bevacizumab have been inconsistent [26, 27]. Our study revealed a trend toward lower pathological complete response rate in patients with CD34 upregulation after concurrent chemoradiotherapy. The increase in CD34 expression after concurrent chemoradiotherapy might represent a response of the tumor to treatment. Given the small number of patients in our study, the true correlation of these markers with the therapeutic response will require further investigation.

The limitations of this study include small patient number, limited follow-up interval, and retrospective comparison with a previously used supine box technique. Pre-concurrent chemoradiotherapy and post-concurrent chemoradiotherapy tumor tissues were only available from nine patients for evaluation of the therapeutic response using potential biomarkers. All these limitations might bias the comparison and endpoints. Although it had a small number of cases, our study implied that prone-position volumetric modulated arc therapy has less bowel toxicity and effectively controls primary tumor and nodal disease. Consequently, a prospective trial of this method (the second cohort) has been initiated in our institution. Serial magnetic resonance imaging, which was associated with histopathological responses [28], was also been performed in the trial. Further patient enrollment and follow-up are needed to confirm our early clinical success.

## Conclusions

Using volumetric modulated arc therapy in the prone position combined with bevacizumab or other novel targeted agents for locally advanced rectal cancer treatment is feasible and safe, achieves a satisfactory pathological response and preliminary disease control, and helps reduce bowel toxicity.

## Abbreviations

FOLFOX: (leucovorin, fluorouracil, and oxaliplatin)
N: node
T: tumor.
